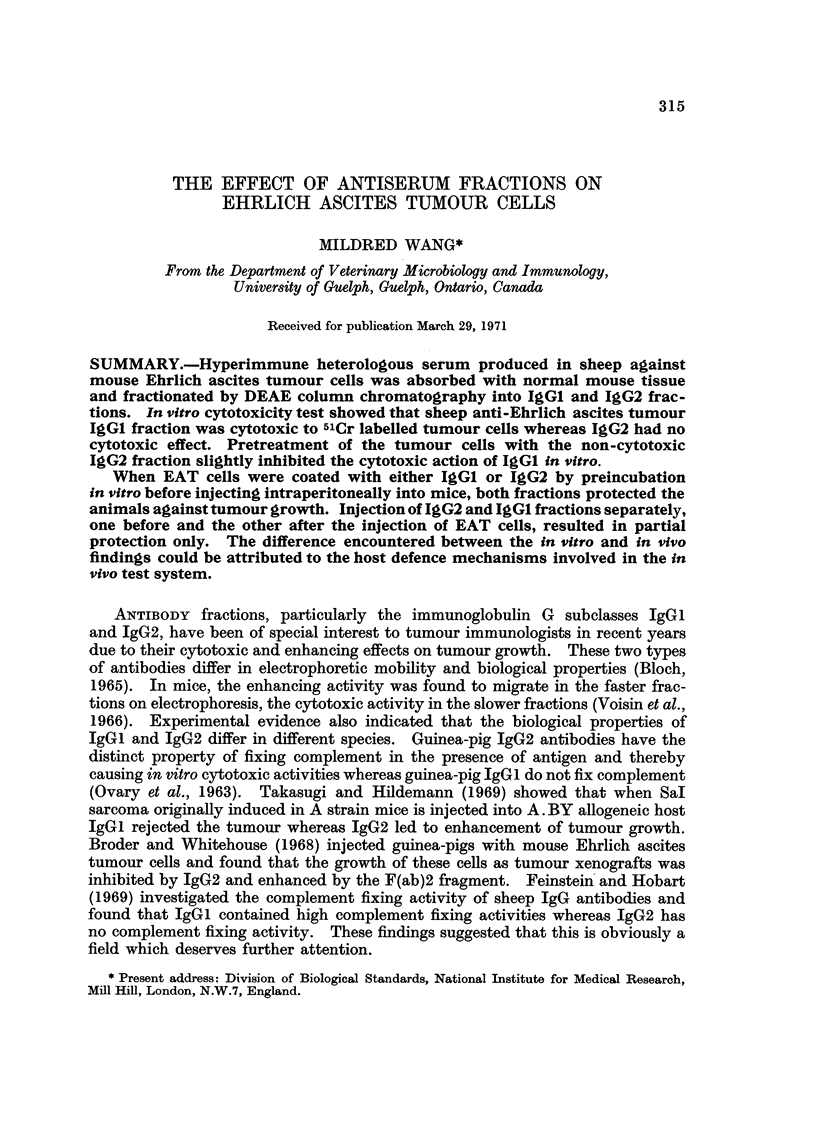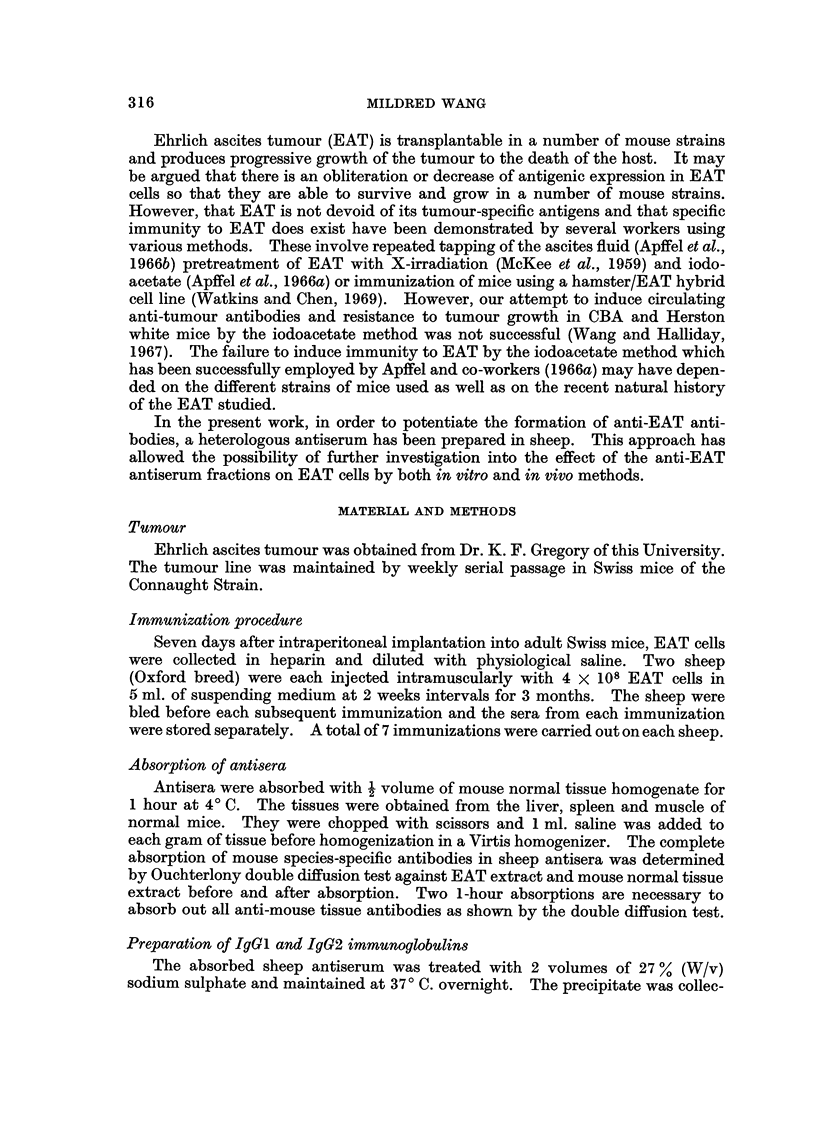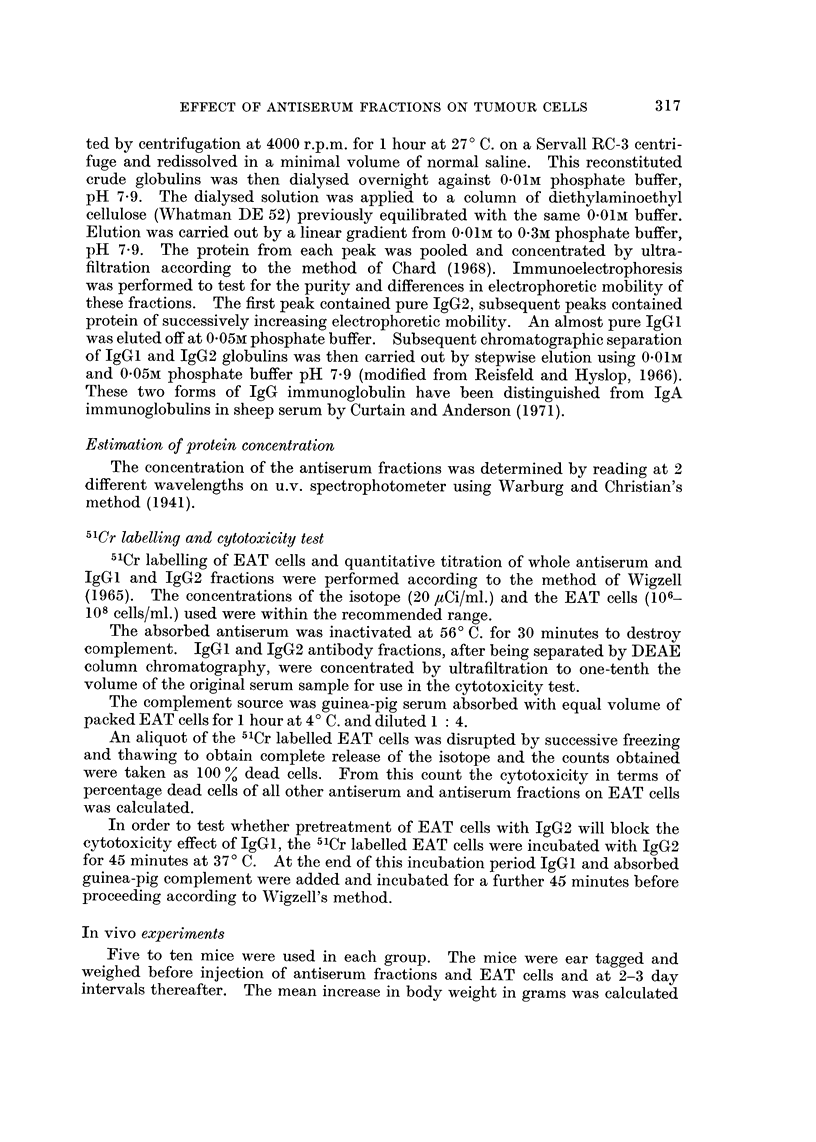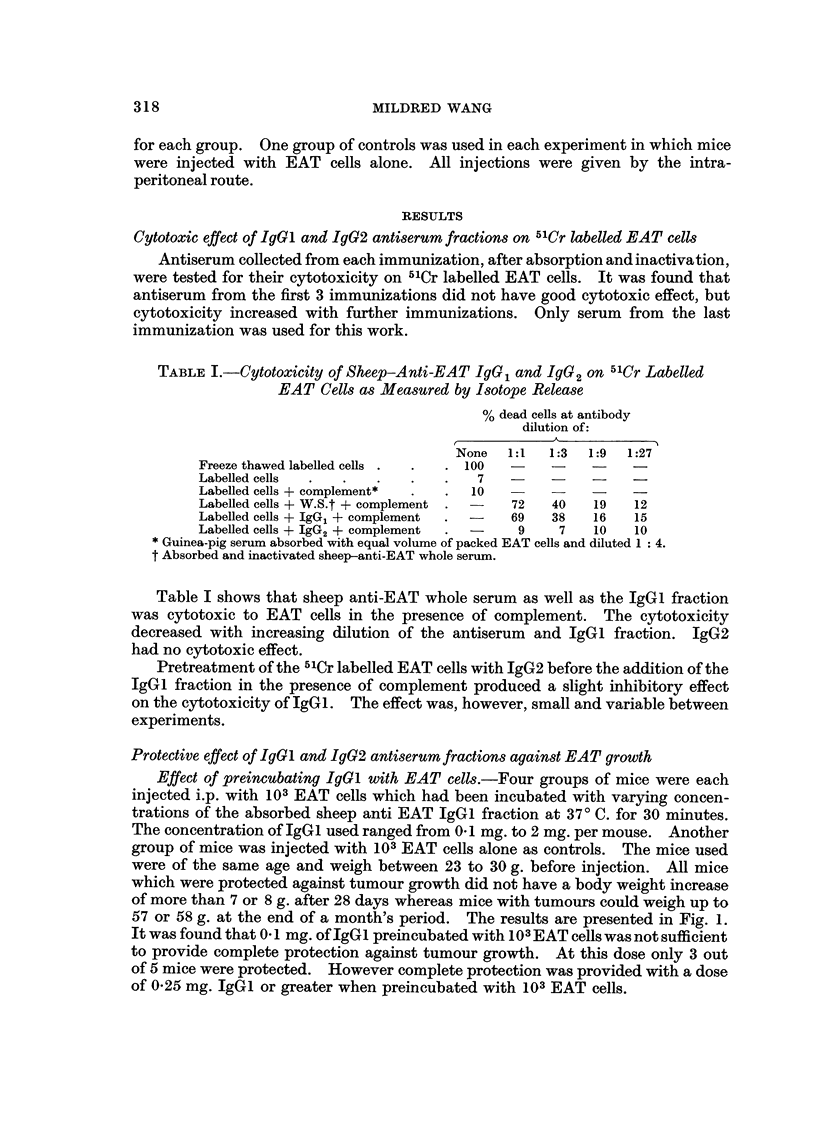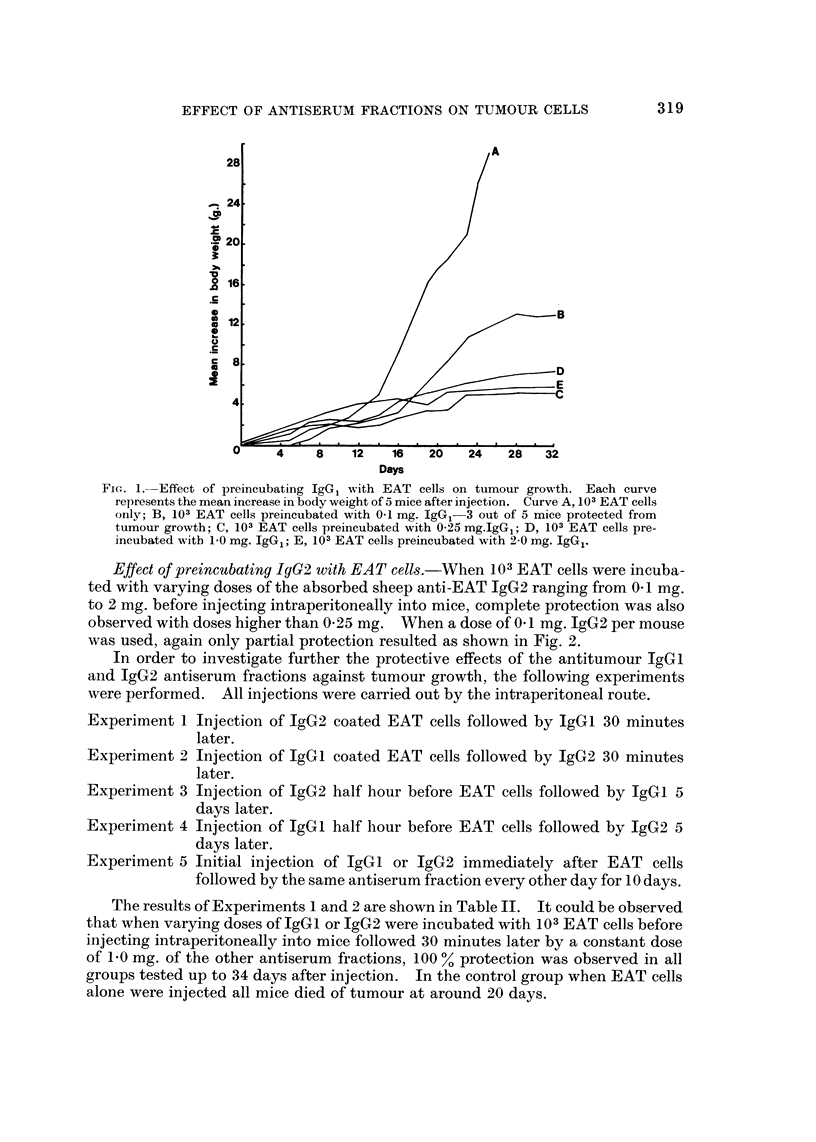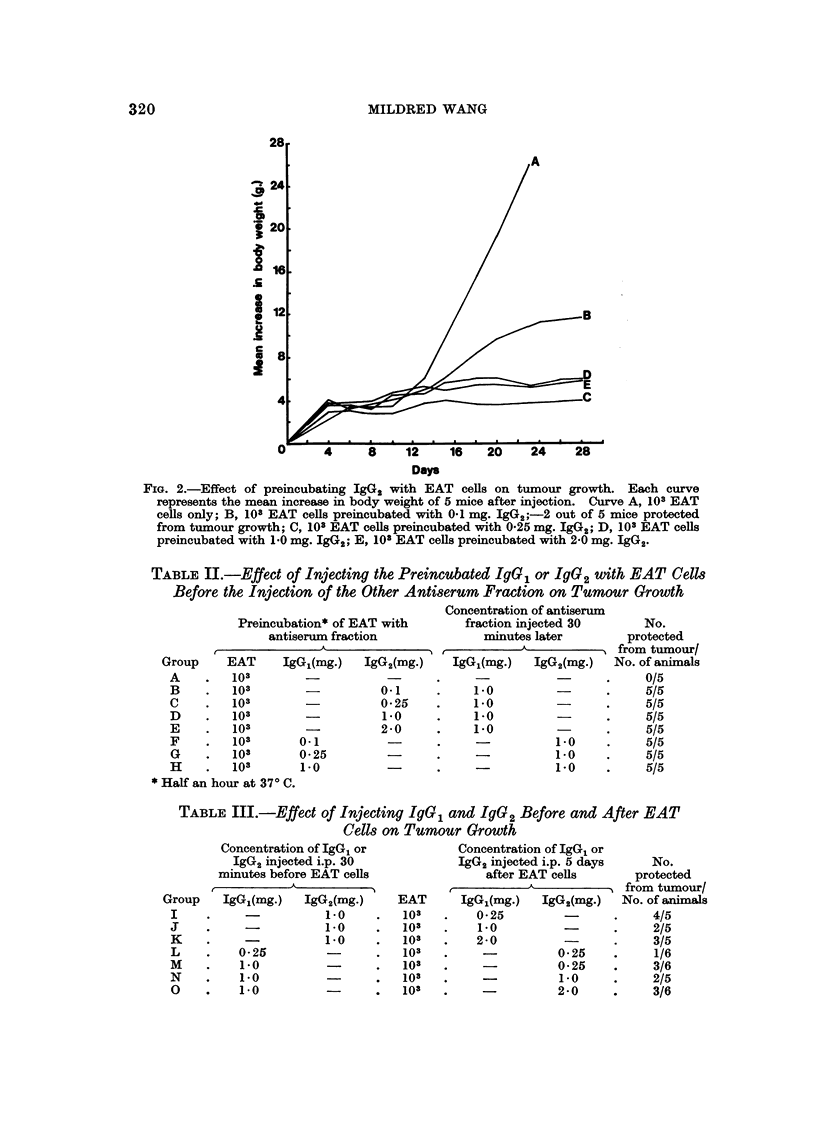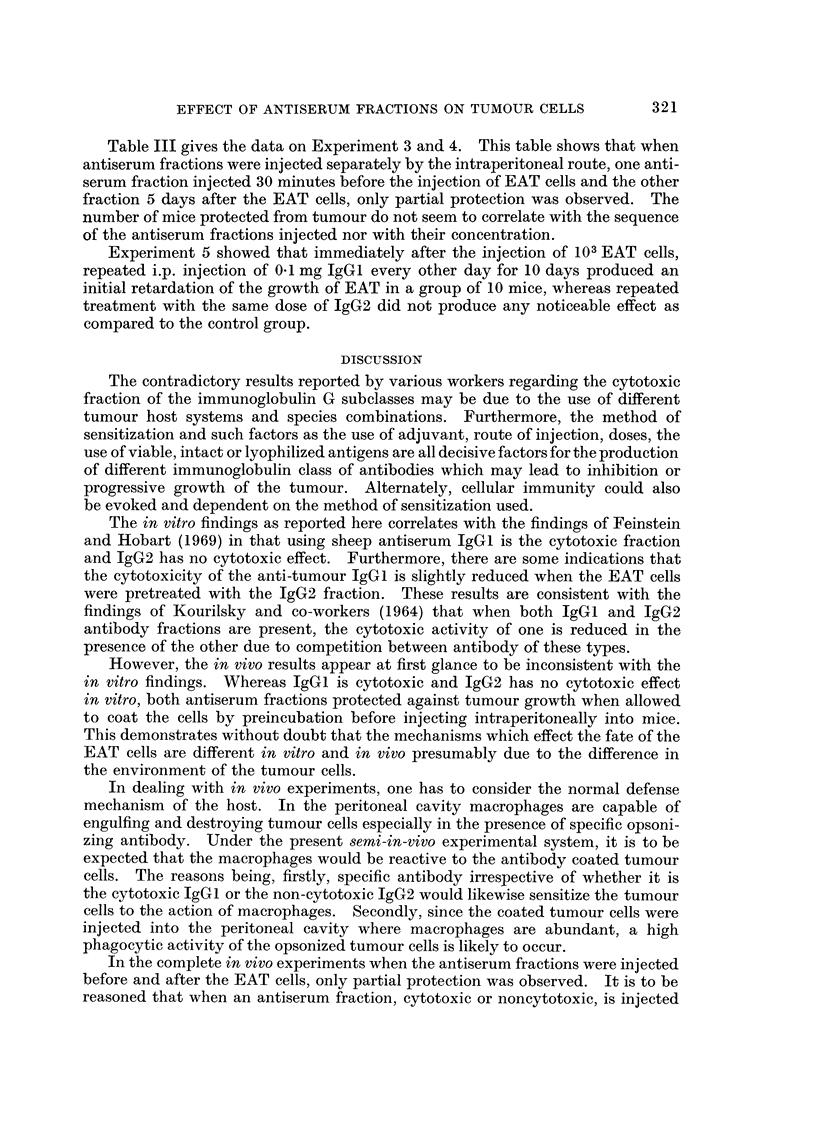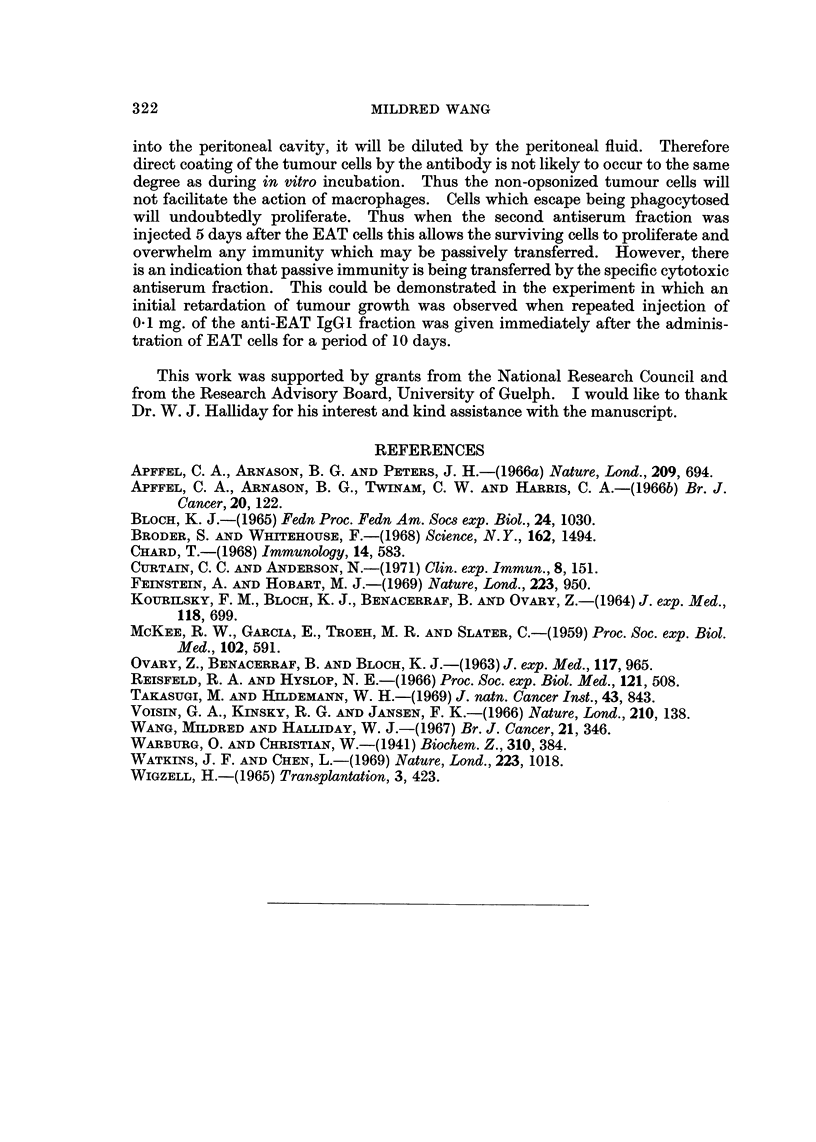# The Effect of Antiserum Fractions on Ehrlich Ascites Tumour Cells

**DOI:** 10.1038/bjc.1971.41

**Published:** 1971-06

**Authors:** Mildred Wang

## Abstract

Hyperimmune heterologous serum produced in sheep against mouse Ehrlich ascites tumour cells was absorbed with normal mouse tissue and fractionated by DEAE column chromatography into IgG1 and IgG2 fractions. *In vitro* cytotoxicity test showed that sheep anti-Ehrlich ascites tumour IgGl fraction was cytotoxic to ^51^Cr labelled tumour cells whereas IgG2 had no cytotoxic effect. Pretreatment of the tumour cells with the non-cytotoxic IgG2 fraction slightly inhibited the cytotoxic action of IgG1 *in vitro*.

When EAT cells were coated with either IgG1 or IgG2 by preincubation *in vitro* before injecting intraperitoneally into mice, both fractions protected the animals against tumour growth. Injection of IgG2 and IgGl fractions separately, one before and the other after the injection of EAT cells, resulted in partial protection only. The difference encountered between the *in vitro* and *in vivo* findings could be attributed to the host defence mechanisms involved in the *in vivo* test system.


### THE EFFECT OF ANTISERUM FRACTIONS ON EHRLICH ASCITES TUMOUR CELLS

MILDRED WANG*

*From the Department of Veterinary Microbiology and Immunology, University of Guelph, Guelph, Ontario, Canada*

Received for publication March 29, 1971

**SUMMARY.—Hyperimmune heterologous serum produced in sheep against mouse Ehrlich ascites tumour cells was absorbed with normal mouse tissue and fractionated by DEAE column chromatography into IgG1 and IgG2 fractions. *In vitro* cytotoxicity test showed that sheep anti-Ehrlich ascites tumour IgG1 fraction was cytotoxic to $^{51}Cr$ labelled tumour cells whereas IgG2 had no cytotoxic effect. Pretreatment of the tumour cells with the non-cytotoxic IgG2 fraction slightly inhibited the cytotoxic action of IgG1 *in vitro*.**

**When EAT cells were coated with either IgG1 or IgG2 by preincubation *in vitro* before injecting intraperitoneally into mice, both fractions protected the animals against tumour growth. Injection of IgG2 and IgG1 fractions separately, one before and the other after the injection of EAT cells, resulted in partial protection only. The difference encountered between the *in vitro* and *in vivo* findings could be attributed to the host defence mechanisms involved in the *in vivo* test system.**

ANTIBODY fractions, particularly the immunoglobulin G subclasses IgG1 and IgG2, have been of special interest to tumour immunologists in recent years due to their cytotoxic and enhancing effects on tumour growth. These two types of antibodies differ in electrophoretic mobility and biological properties (Bloch, 1965). In mice, the enhancing activity was found to migrate in the faster fractions on electrophoresis, the cytotoxic activity in the slower fractions (Voisin *et al.*, 1966). Experimental evidence also indicated that the biological properties of IgG1 and IgG2 differ in different species. Guinea-pig IgG2 antibodies have the distinct property of fixing complement in the presence of antigen and thereby causing *in vitro* cytotoxic activities whereas guinea-pig IgG1 do not fix complement (Ovary *et al.*, 1963). Takasugi and Hildemann (1969) showed that when SaI sarcoma originally induced in A strain mice is injected into A.BY allogeneic host IgG1 rejected the tumour whereas IgG2 led to enhancement of tumour growth. Broder and Whitehouse (1968) injected guinea-pigs with mouse Ehrlich ascites tumour cells and found that the growth of these cells as tumour xenografts was inhibited by IgG2 and enhanced by the F(ab)2 fragment. Feinstein and Hobart (1969) investigated the complement fixing activity of sheep IgG antibodies and found that IgG1 contained high complement fixing activities whereas IgG2 has no complement fixing activity. These findings suggested that this is obviously a field which deserves further attention.

* Present address: Division of Biological Standards, National Institute for Medical Research, Mill Hill, London, N.W.7, England.

Ehrlich ascites tumour (EAT) is transplantable in a number of mouse strains and produces progressive growth of the tumour to the death of the host. It may be argued that there is an obliteration or decrease of antigenic expression in EAT cells so that they are able to survive and grow in a number of mouse strains. However, that EAT is not devoid of its tumour-specific antigens and that specific immunity to EAT does exist have been demonstrated by several workers using various methods. These involve repeated tapping of the ascites fluid (Apffel *et al.*, 1966*b*) pretreatment of EAT with X-irradiation (McKee *et al.*, 1959) and iodoacetate (Apffel *et al.*, 1966*a*) or immunization of mice using a hamster/EAT hybrid cell line (Watkins and Chen, 1969). However, our attempt to induce circulating anti-tumour antibodies and resistance to tumour growth in CBA and Herston white mice by the iodoacetate method was not successful (Wang and Halliday, 1967). The failure to induce immunity to EAT by the iodoacetate method which has been successfully employed by Apffel and co-workers (1966*a*) may have depended on the different strains of mice used as well as on the recent natural history of the EAT studied.

In the present work, in order to potentiate the formation of anti-EAT antibodies, a heterologous antiserum has been prepared in sheep. This approach has allowed the possibility of further investigation into the effect of the anti-EAT antiserum fractions on EAT cells by both *in vitro* and *in vivo* methods.

### MATERIAL AND METHODS

### *Tumour*

Ehrlich ascites tumour was obtained from Dr. K. F. Gregory of this University. The tumour line was maintained by weekly serial passage in Swiss mice of the Connaught Strain.

### *Immunization procedure*

Seven days after intraperitoneal implantation into adult Swiss mice, EAT cells were collected in heparin and diluted with physiological saline. Two sheep (Oxford breed) were each injected intramuscularly with $4 \times 10^8$ EAT cells in 5 ml. of suspending medium at 2 weeks intervals for 3 months. The sheep were bled before each subsequent immunization and the sera from each immunization were stored separately. A total of 7 immunizations were carried out on each sheep.

### *Absorption of antisera*

Antisera were absorbed with $\frac{1}{2}$ volume of mouse normal tissue homogenate for 1 hour at 4° C. The tissues were obtained from the liver, spleen and muscle of normal mice. They were chopped with scissors and 1 ml. saline was added to each gram of tissue before homogenization in a Virtis homogenizer. The complete absorption of mouse species-specific antibodies in sheep antisera was determined by Ouchterlony double diffusion test against EAT extract and mouse normal tissue extract before and after absorption. Two 1-hour absorptions are necessary to absorb out all anti-mouse tissue antibodies as shown by the double diffusion test.

### *Preparation of IgG*1 *and IgG*2 *immunoglobulins*

The absorbed sheep antiserum was treated with 2 volumes of 27% (W/v) sodium sulphate and maintained at 37° C. overnight. The precipitate was collected by centrifugation at 4000 r.p.m. for 1 hour at 27° C. on a Servall RC-3 centrifuge and redissolved in a minimal volume of normal saline. This reconstituted crude globulins was then dialysed overnight against 0·01M phosphate buffer, pH 7·9. The dialysed solution was applied to a column of diethylaminoethyl cellulose (Whatman DE 52) previously equilibrated with the same 0·01M buffer. Elution was carried out by a linear gradient from 0·01M to 0·3M phosphate buffer, pH 7·9. The protein from each peak was pooled and concentrated by ultrafiltration according to the method of Chard (1968). Immunoelectrophoresis was performed to test for the purity and differences in electrophoretic mobility of these fractions. The first peak contained pure IgG2, subsequent peaks contained protein of successively increasing electrophoretic mobility. An almost pure IgG1 was eluted off at 0·05M phosphate buffer. Subsequent chromatographic separation of IgG1 and IgG2 globulins was then carried out by stepwise elution using 0·01M and 0·05M phosphate buffer pH 7·9 (modified from Reisfeld and Hyslop, 1966). These two forms of IgG immunoglobulin have been distinguished from IgA immunoglobulins in sheep serum by Curtain and Anderson (1971).

*Estimation of protein concentration*

The concentration of the antiserum fractions was determined by reading at 2 different wavelengths on u.v. spectrophotometer using Warburg and Christian's method (1941).

*$^{51}$Cr labelling and cytotoxicity test*

$^{51}$Cr labelling of EAT cells and quantitative titration of whole antiserum and IgG1 and IgG2 fractions were performed according to the method of Wigzell (1965). The concentrations of the isotope (20 $\mu$Ci/ml.) and the EAT cells ($10^6$–$10^8$ cells/ml.) used were within the recommended range.

The absorbed antiserum was inactivated at 56° C. for 30 minutes to destroy complement. IgG1 and IgG2 antibody fractions, after being separated by DEAE column chromatography, were concentrated by ultrafiltration to one-tenth the volume of the original serum sample for use in the cytotoxicity test.

The complement source was guinea-pig serum absorbed with equal volume of packed EAT cells for 1 hour at 4° C. and diluted 1 : 4.

An aliquot of the $^{51}$Cr labelled EAT cells was disrupted by successive freezing and thawing to obtain complete release of the isotope and the counts obtained were taken as 100% dead cells. From this count the cytotoxicity in terms of percentage dead cells of all other antiserum and antiserum fractions on EAT cells was calculated.

In order to test whether pretreatment of EAT cells with IgG2 will block the cytotoxicity effect of IgG1, the $^{51}$Cr labelled EAT cells were incubated with IgG2 for 45 minutes at 37° C. At the end of this incubation period IgG1 and absorbed guinea-pig complement were added and incubated for a further 45 minutes before proceeding according to Wigzell's method.

In vivo *experiments*

Five to ten mice were used in each group. The mice were ear tagged and weighed before injection of antiserum fractions and EAT cells and at 2–3 day intervals thereafter. The mean increase in body weight in grams was calculated for each group. One group of controls was used in each experiment in which mice were injected with EAT cells alone. All injections were given by the intraperitoneal route.

### RESULTS

*Cytotoxic effect of IgG1 and IgG2 antiserum fractions on $^{51}Cr$ labelled EAT cells*

Antiserum collected from each immunization, after absorption and inactivation, were tested for their cytotoxicity on $^{51}$Cr labelled EAT cells. It was found that antiserum from the first 3 immunizations did not have good cytotoxic effect, but cytotoxicity increased with further immunizations. Only serum from the last immunization was used for this work.

TABLE I.—*Cytotoxicity of Sheep-Anti-EAT $IgG_1$ and $IgG_2$ on $^{51}Cr$ Labelled EAT Cells as Measured by Isotope Release*

| | % dead cells at antibody dilution of: | | | | |
|---|---|---|---|---|---|
| | None | 1:1 | 1:3 | 1:9 | 1:27 |
| Freeze thawed labelled cells | 100 | — | — | — | — |
| Labelled cells | 7 | — | — | — | — |
| Labelled cells + complement* | 10 | — | — | — | — |
| Labelled cells + W.S.† + complement | — | 72 | 40 | 19 | 12 |
| Labelled cells + $IgG_1$ + complement | — | 69 | 38 | 16 | 15 |
| Labelled cells + $IgG_2$ + complement | — | 9 | 7 | 10 | 10 |

* Guinea-pig serum absorbed with equal volume of packed EAT cells and diluted 1 : 4.
† Absorbed and inactivated sheep–anti-EAT whole serum.

Table I shows that sheep anti-EAT whole serum as well as the IgG1 fraction was cytotoxic to EAT cells in the presence of complement. The cytotoxicity decreased with increasing dilution of the antiserum and IgG1 fraction. IgG2 had no cytotoxic effect.

Pretreatment of the $^{51}$Cr labelled EAT cells with IgG2 before the addition of the IgG1 fraction in the presence of complement produced a slight inhibitory effect on the cytotoxicity of IgG1. The effect was, however, small and variable between experiments.

*Protective effect of IgG1 and IgG2 antiserum fractions against EAT growth*

*Effect of preincubating IgG1 with EAT cells.*—Four groups of mice were each injected i.p. with $10^3$ EAT cells which had been incubated with varying concentrations of the absorbed sheep anti EAT IgG1 fraction at 37° C. for 30 minutes. The concentration of IgG1 used ranged from 0·1 mg. to 2 mg. per mouse. Another group of mice was injected with $10^3$ EAT cells alone as controls. The mice used were of the same age and weigh between 23 to 30 g. before injection. All mice which were protected against tumour growth did not have a body weight increase of more than 7 or 8 g. after 28 days whereas mice with tumours could weigh up to 57 or 58 g. at the end of a month's period. The results are presented in Fig. 1. It was found that 0·1 mg. of IgG1 preincubated with $10^3$ EAT cells was not sufficient to provide complete protection against tumour growth. At this dose only 3 out of 5 mice were protected. However complete protection was provided with a dose of 0·25 mg. IgG1 or greater when preincubated with $10^3$ EAT cells.

FIG. 1.—Effect of preincubating $IgG_1$ with EAT cells on tumour growth. Each curve represents the mean increase in body weight of 5 mice after injection. Curve A, $10^3$ EAT cells only; B, $10^3$ EAT cells preincubated with 0·1 mg. $IgG_1$—3 out of 5 mice protected from tumour growth; C, $10^3$ EAT cells preincubated with 0·25 mg.$IgG_1$; D, $10^3$ EAT cells preincubated with 1·0 mg. $IgG_1$; E, $10^3$ EAT cells preincubated with 2·0 mg. $IgG_1$.

*Effect of preincubating IgG2 with EAT cells.*—When $10^3$ EAT cells were incubated with varying doses of the absorbed sheep anti-EAT IgG2 ranging from 0·1 mg. to 2 mg. before injecting intraperitoneally into mice, complete protection was also observed with doses higher than 0·25 mg. When a dose of 0·1 mg. IgG2 per mouse was used, again only partial protection resulted as shown in Fig. 2.

In order to investigate further the protective effects of the antitumour IgG1 and IgG2 antiserum fractions against tumour growth, the following experiments were performed. All injections were carried out by the intraperitoneal route.

Experiment 1 Injection of IgG2 coated EAT cells followed by IgG1 30 minutes later.

Experiment 2 Injection of IgG1 coated EAT cells followed by IgG2 30 minutes later.

Experiment 3 Injection of IgG2 half hour before EAT cells followed by IgG1 5 days later.

Experiment 4 Injection of IgG1 half hour before EAT cells followed by IgG2 5 days later.

Experiment 5 Initial injection of IgG1 or IgG2 immediately after EAT cells followed by the same antiserum fraction every other day for 10 days.

The results of Experiments 1 and 2 are shown in Table II. It could be observed that when varying doses of IgG1 or IgG2 were incubated with $10^3$ EAT cells before injecting intraperitoneally into mice followed 30 minutes later by a constant dose of 1·0 mg. of the other antiserum fractions, 100 % protection was observed in all groups tested up to 34 days after injection. In the control group when EAT cells alone were injected all mice died of tumour at around 20 days.

FIG. 2.—Effect of preincubating $IgG_2$ with EAT cells on tumour growth. Each curve represents the mean increase in body weight of 5 mice after injection. Curve A, $10^3$ EAT cells only; B, $10^3$ EAT cells preincubated with 0·1 mg. $IgG_2$;—2 out of 5 mice protected from tumour growth; C, $10^3$ EAT cells preincubated with 0·25 mg. $IgG_2$; D, $10^3$ EAT cells preincubated with 1·0 mg. $IgG_2$; E, $10^3$ EAT cells preincubated with 2·0 mg. $IgG_2$.

TABLE II.—*Effect of Injecting the Preincubated $IgG_1$ or $IgG_2$ with EAT Cells Before the Injection of the Other Antiserum Fraction on Tumour Growth*

| | Preincubation* of EAT with antiserum fraction | | | Concentration of antiserum fraction injected 30 minutes later | | No. protected from tumour/ |
|---|---|---|---|---|---|---|
| Group | EAT | $IgG_1$(mg.) | $IgG_2$(mg.) | $IgG_1$(mg.) | $IgG_2$(mg.) | No. of animals |
| A | $10^3$ | — | — | — | — | 0/5 |
| B | $10^3$ | — | 0·1 | 1·0 | — | 5/5 |
| C | $10^3$ | — | 0·25 | 1·0 | — | 5/5 |
| D | $10^3$ | — | 1·0 | 1·0 | — | 5/5 |
| E | $10^3$ | — | 2·0 | 1·0 | — | 5/5 |
| F | $10^3$ | 0·1 | — | — | 1·0 | 5/5 |
| G | $10^3$ | 0·25 | — | — | 1·0 | 5/5 |
| H | $10^3$ | 1·0 | — | — | 1·0 | 5/5 |

* Half an hour at 37° C.

TABLE III.—*Effect of Injecting $IgG_1$ and $IgG_2$ Before and After EAT Cells on Tumour Growth*

| | Concentration of $IgG_1$ or $IgG_2$ injected i.p. 30 minutes before EAT cells | | | Concentration of $IgG_1$ or $IgG_2$ injected i.p. 5 days after EAT cells | | No. protected from tumour/ |
|---|---|---|---|---|---|---|
| Group | $IgG_1$(mg.) | $IgG_2$(mg.) | EAT | $IgG_1$(mg.) | $IgG_2$(mg.) | No. of animals |
| I | — | 1·0 | $10^3$ | 0·25 | — | 4/5 |
| J | — | 1·0 | $10^3$ | 1·0 | — | 2/5 |
| K | — | 1·0 | $10^3$ | 2·0 | — | 3/5 |
| L | 0·25 | — | $10^3$ | — | 0·25 | 1/6 |
| M | 1·0 | — | $10^3$ | — | 0·25 | 3/6 |
| N | 1·0 | — | $10^3$ | — | 1·0 | 2/5 |
| O | 1·0 | — | $10^3$ | — | 2·0 | 3/6 |

Table III gives the data on Experiment 3 and 4. This table shows that when antiserum fractions were injected separately by the intraperitoneal route, one antiserum fraction injected 30 minutes before the injection of EAT cells and the other fraction 5 days after the EAT cells, only partial protection was observed. The number of mice protected from tumour do not seem to correlate with the sequence of the antiserum fractions injected nor with their concentration.

Experiment 5 showed that immediately after the injection of $10^3$ EAT cells, repeated i.p. injection of 0·1 mg IgG1 every other day for 10 days produced an initial retardation of the growth of EAT in a group of 10 mice, whereas repeated treatment with the same dose of IgG2 did not produce any noticeable effect as compared to the control group.

### DISCUSSION

The contradictory results reported by various workers regarding the cytotoxic fraction of the immunoglobulin G subclasses may be due to the use of different tumour host systems and species combinations. Furthermore, the method of sensitization and such factors as the use of adjuvant, route of injection, doses, the use of viable, intact or lyophilized antigens are all decisive factors for the production of different immunoglobulin class of antibodies which may lead to inhibition or progressive growth of the tumour. Alternately, cellular immunity could also be evoked and dependent on the method of sensitization used.

The *in vitro* findings as reported here correlates with the findings of Feinstein and Hobart (1969) in that using sheep antiserum IgG1 is the cytotoxic fraction and IgG2 has no cytotoxic effect. Furthermore, there are some indications that the cytotoxicity of the anti-tumour IgG1 is slightly reduced when the EAT cells were pretreated with the IgG2 fraction. These results are consistent with the findings of Kourilsky and co-workers (1964) that when both IgG1 and IgG2 antibody fractions are present, the cytotoxic activity of one is reduced in the presence of the other due to competition between antibody of these types.

However, the *in vivo* results appear at first glance to be inconsistent with the *in vitro* findings. Whereas IgG1 is cytotoxic and IgG2 has no cytotoxic effect *in vitro*, both antiserum fractions protected against tumour growth when allowed to coat the cells by preincubation before injecting intraperitoneally into mice. This demonstrates without doubt that the mechanisms which effect the fate of the EAT cells are different *in vitro* and *in vivo* presumably due to the difference in the environment of the tumour cells.

In dealing with *in vivo* experiments, one has to consider the normal defense mechanism of the host. In the peritoneal cavity macrophages are capable of engulfing and destroying tumour cells especially in the presence of specific opsonizing antibody. Under the present *semi-in-vivo* experimental system, it is to be expected that the macrophages would be reactive to the antibody coated tumour cells. The reasons being, firstly, specific antibody irrespective of whether it is the cytotoxic IgG1 or the non-cytotoxic IgG2 would likewise sensitize the tumour cells to the action of macrophages. Secondly, since the coated tumour cells were injected into the peritoneal cavity where macrophages are abundant, a high phagocytic activity of the opsonized tumour cells is likely to occur.

In the complete *in vivo* experiments when the antiserum fractions were injected before and after the EAT cells, only partial protection was observed. It is to be reasoned that when an antiserum fraction, cytotoxic or noncytotoxic, is injected into the peritoneal cavity, it will be diluted by the peritoneal fluid. Therefore direct coating of the tumour cells by the antibody is not likely to occur to the same degree as during *in vitro* incubation. Thus the non-opsonized tumour cells will not facilitate the action of macrophages. Cells which escape being phagocytosed will undoubtedly proliferate. Thus when the second antiserum fraction was injected 5 days after the EAT cells this allows the surviving cells to proliferate and overwhelm any immunity which may be passively transferred. However, there is an indication that passive immunity is being transferred by the specific cytotoxic antiserum fraction. This could be demonstrated in the experiment in which an initial retardation of tumour growth was observed when repeated injection of 0·1 mg. of the anti-EAT IgG1 fraction was given immediately after the administration of EAT cells for a period of 10 days.

This work was supported by grants from the National Research Council and from the Research Advisory Board, University of Guelph. I would like to thank Dr. W. J. Halliday for his interest and kind assistance with the manuscript.

### REFERENCES

Apffel, C. A., Arnason, B. G. and Peters, J. H.—(1966*a*) *Nature, Lond.*, **209**, 694.
Apffel, C. A., Arnason, B. G., Twinam, C. W. and Harris, C. A.—(1966*b*) *Br. J. Cancer*, **20**, 122.
Bloch, K. J.—(1965) *Fedn Proc. Fedn Am. Socs exp. Biol.*, **24**, 1030.
Broder, S. and Whitehouse, F.—(1968) *Science, N.Y.*, **162**, 1494.
Chard, T.—(1968) *Immunology*, **14**, 583.
Curtain, C. C. and Anderson, N.—(1971) *Clin. exp. Immun.*, **8**, 151.
Feinstein, A. and Hobart, M. J.—(1969) *Nature, Lond.*, **223**, 950.
Kourilsky, F. M., Bloch, K. J., Benacerraf, B. and Ovary, Z.—(1964) *J. exp. Med.*, **118**, 699.
McKee, R. W., Garcia, E., Troeh, M. R. and Slater, C.—(1959) *Proc. Soc. exp. Biol. Med.*, **102**, 591.
Ovary, Z., Benacerraf, B. and Bloch, K. J.—(1963) *J. exp. Med.*, **117**, 965.
Reisfeld, R. A. and Hyslop, N. E.—(1966) *Proc. Soc. exp. Biol. Med.*, **121**, 508.
Takasugi, M. and Hildemann, W. H.—(1969) *J. natn. Cancer Inst.*, **43**, 843.
Voisin, G. A., Kinsky, R. G. and Jansen, F. K.—(1966) *Nature, Lond.*, **210**, 138.
Wang, Mildred and Halliday, W. J.—(1967) *Br. J. Cancer*, **21**, 346.
Warburg, O. and Christian, W.—(1941) *Biochem. Z.*, **310**, 384.
Watkins, J. F. and Chen, L.—(1969) *Nature, Lond.*, **223**, 1018.
Wigzell, H.—(1965) *Transplantation*, **3**, 423.